# Neonatal Brain Tumors: A Review

**DOI:** 10.21699/jns.v6i2.579

**Published:** 2017-04-15

**Authors:** Shaam Bodeliwala, Vikas Kumar, Daljit Singh

**Affiliations:** Department of Neurosurgery, G. B. Pant Institute of Postgraduate Education and Research (GIPMER), New Delhi.

**Keywords:** Tumors, Brain, Neonate

## Abstract

Brain tumors in neonatal age group is uncommon comparing with older children and adults. In older children brain tumors are commonly infratentorial, where as in neonates, they are supratentorial. Though extracranial tumors are commoner in neonates, brain tumors cause 5-20% deaths approximately. We are presenting a review on brain tumors in neonates.

## Incidence

Central nervous system malignancy is the third most common pediatric malignancy in India [1,2]. Fortunately, incidence of neonatal brain tumors (NBT) is very low comprising <1% of pediatric CNS malignancy[1], the exact incidence of NBT is not known in India due to lack of complete registration and data collection. 

## Classifications

Histological types of common neonatal brain tumors are different from older children and adults. Types of NBTs are teratomas, astrocytomas, embryonal tumors, choroid plexus tumors, craniopharyngiomas, gangliogliomas, ependymal and meningeal tumors and other miscellaneous tumors. Among them teratoma is being the commonest tumor comprising of almost one third of cases[1]. Indian neonatal brain tumor incidence in different types is not specified, but astrocytoma (34.7%) being the most common pediatric brain tumor followed by medulloblastoma and primitive neuroectodermal tumors (22.4%)[3]. Another single center study from southern India mentioned incidence of the most common pediatric brain tumor as astrocytoma 47.3% followed by medulloblastoma 11.4% and craniopharyngioma 9.7%[4]. 

## Clinical features

NBTs are mainly classified into three groups according to presentation: prenatal, perinatal and postnatal. Since last two decades, use of advanced technology in antenatal imaging has increased incidence of prenatal diagnosis of NBTs. Antenatal ultrasonography is the main stay of diagnosis. Increased head size, large for gestational age uterus, polyhydramnios, hydrops, and hydrocephalus are initial presenting findings in antenatal ultrasonography. Ultrasonography also finds macrocephaly, hydrocephalus, an abnormal intracranial echogenicity or features suggestive of intracranial mass. Nowadays, use of fetal magnetic resonance imaging (MRI) in suspicious cases has increased specificity in the diagnosis of brain tumors. Others can present with obstructed labor or dystocia due to cephalo-pelvic disproportion owing to large head. It may cause stillbirth or premature labor. Postnatally, neonates present with large head, symptoms of raised intracranial pressure, bulging fontanels, irritability, drowsiness, morning vomiting, failure to thrive, apnoeic episodes, neurological deficits or seizures [1,5].

Volpe [6] divided NBTs in four groups: (1) severe macrocrania caused by gigantic tumors leading to cephalo-pelvic disporportion, dystocia, stillbirth, or premature labor; (2) hydrocephalus with a large head and bulging fontanel; (3) specific neurologic findings according to the type and site of the lesion (e.g. hemiparesis, or quadri-paresis, seizures, cranial nerve abnormalities, and increased intracranial pressure); (4) sudden intracranial hemorrhage (8-18%).

Large head size or macrocephaly (28.7-60%) [1,7] is the commonest finding in such neonates, while hydrocephalus (17.3%) [1] is the second most common. Macrocephaly can be due to tumor itself or hydrocephalus. Hydrocephalus commonly occurs with compression of ventricles by tumor or hemorrhage from the tumor or hypersecretion of cerebrospinal fluid (CSF) in case of choroid plexus tumors. Seizure is the rare presentation but may be present in some cases (10–15%) [5]. Intra-ventricular hemorrhage is rare but has been reported in few cases of astrocytoma [3]. 

## Radiology 

**Figure 1:**
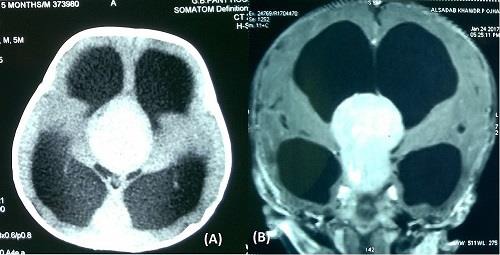
figures, computed tomography (A) and contrast T1-weighted magnetic resonance image (B) of a 4 months old child showing a tumor in suprachiasmatic region extending into third ventricle, which is homogeneously contrast enhancing. These radi-ological features are suggestive of optico-chiasmatic glioma.

**Figure 2:**
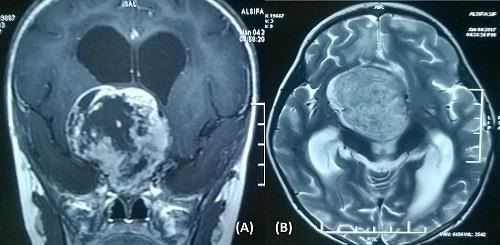
figures, contrast T1-weighted (A) and T2-weighted (B) magnetic resonance images of 2 months old child showing a heter-ogeneously enhancing solid-cystic mass extending from suprachi-asmtic region to third ventricle. Histopathological diagnosis was pilocytic astrocytoma.

**Figure 3:**
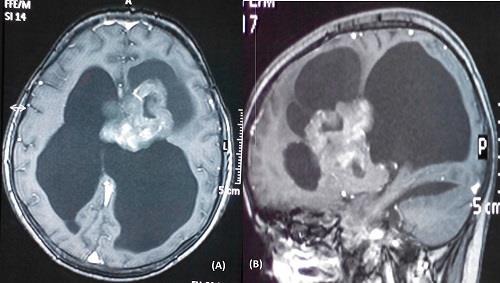
figures, contrast T1-weighted axial (A) and sagittal (B) magnetic resonance images of a child showing an intraventricular contrast enhancing lesion with hydrocephalus, features compati-ble with of choroid plexus papilloma.

**Figure 4:**
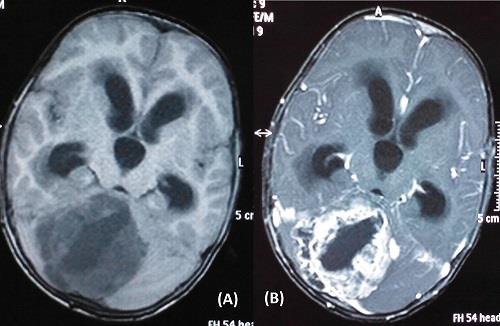
Non-contrast (A) and contrast enhanced (B)T1-weighted magnetic resonance images of a patient showing solid-cystic mass with heterogeneous contrast enhancement and intensity sugges-tive of medulloblastoma.

Magnetic resonance imaging (MRI) is the choice of investigation (Fig.1-4). Screening of the entire neuraxis is necessary as germ cell tumours, embryonal tumors and occasionally pilocytic (benign) lesions have a propensity to metastasise through CSF and create drop metastasis [5]. Some radiological features of common neonatal brain tumors are summarized in table 1. Disadvantage of MRI is that it takes several minutes and neonates are required to stay still. Often neonates are subjected to general anesthesia if sedatives do not serve the purpose. MRI delineates anatomy of surrounding structures and extent of the tumor very well but does not show calcification present in tumors, which is helpful to differentiate subtypes of glioma-oligodendroglioma or ganglioglioma. Computed tomography shows calcification better but uses high dose of ionizing radiation. Ultrasonography has the limited role in postnatal period. For the tumors in the pineal region, biomarkers like alpha-feto protein (AFP), human beta-chorionic gonadotropin (hCG) levels are of help in diagnosis, as they are raised in germ cell tumors, choriocarcinoma and yolk cell tumors.

Antenatal diagnosis of congenital brain tumors is possible by antenatal ultrasonography and fetal MRI. Fetal MRI can delineate tumor and surrounding anatomy but it is indicated after ultrasound in third trimester. Safety of MRI has not been established in second or first trimester. Gadolinium MRI contrast is not recommended during pregnancy. A fast T2-weighted single-shot technique on a 1.5-Tesla machine is recommended for fetal anatomy assessment to minimize the fetal movement artifacts [8]. Caution should be taken while using a 3-Tesla machine in indicated cases.

## Management 

Treatment of neonatal brain tumors have two aspects: surgical resection and adjuvant chemotherapy. Radiotherapy is contraindicated in children less than three years of age due to its exaggerated long term side effects in neonates like developmental retardation, neuro-endocrine abnormalities, the risk of secondary neoplasms in the CNS and premature death [5]. Radiotherapy can be postponed up to three years of age as chemotherapy can buy time.

Surgical resection is the primary treatment of NBTs. It decreases the tumor burden as provides tissue for histological diagnosis. Gross total resection (GTR) is independently correlated with improved survival even in pediatric population [9,10]. Surgical resection can be done by open or endoscopic approach. Endoscopic approach is suitable for intra-ventricular tumors, particularly small tumors. Large tumors or parenchymal tumors are better approached through open surgery as per our experience. Additionally, it can be combined with endoscopic third ventriculostomy (ETV) in patients with obstructive hydrocephalus.

Major hurdle of open surgery in NBTs, particularly for large tumors, is blood loss. Blood loss and tumor location in eloquent area preclude gross total resection [5]. Poor outcome in neonates is due to subtotal resection, high-grade tumor histology and avoidance of radiotherapy [5]. Review of literature did not show relevance of mortality with tumor size in NBTs. 

Many patients present with symptomatic hydrocephalus, they require CSF diversion in form of ventriculo-peritoneal shunt, ETV or external ventricular drainage. Such patients can be underwent CSF diversion procedure in emergency followed by planned surgical tumor resection. Some patients may require a CSF diversion in postoperative period. One study from southern India mentioned that short history, Evan’s index at the time of presentation and need of intraoperative ventricular drainage are positively associated with requirement of CSF shunt following posterior fossa tumor resection in pediatric patients [11].

Adjuvant chemotherapy is the second aspect of treatment of NBTs. Multi-agent chemotherapy had been tried with or without delayed radiotherapy after surgical resection. A German study involving children less than 3 years of anaplastic ependymoma suggested that delaying radiotherapy decreases the survival even after intensive chemotherapy [12]. The prospective multicenter trial HIT 2000 proposed short intensive induction chemotherapy using carboplatin and etoposide followed by tandem-high dose chemotherapy (HDCT) in young children with CNS-PNET/pineoblastomas increased survival benefits [13]. In one American study, second look surgery was done in enrolled patients to achieve the GTR after multi-agent chemotherapy and found induction chemotherapy resulted in devascularization of tumors with volume regression in the majority and subsequently facilitated resection with acceptable intraoperative blood loss. GTR achieved in second look surgery added survival benefit. This concluded that a GTR of infantile brain tumors is one of the most important predictors of outcome [10]. Many studies indicated that tandem HDCT with autologous stem cell transplantation (SCT) may improve survival in young children in malignant brain tumors with an acceptable risk [14,15]. But elaborative studies are required for further implementation.

## Prognosis

Survival in neonatal brain tumors is poor due to less aggressive tumor resection, high grade pathology and avoidance of radiotherapy. Location of the tumor is also an important factor as in eloquent areas surgical resection is difficult, for example brain stem glioma. Median survival in pediatric brain stem glioma is 10 months [16]. Mortality was more than 90% in neonatal brain tumors within 5 years of age [17]. Isaacs reported 15% overall survival in a review of 154 cases (54 were stillborn) diagnosed prenatally [18]. High-grade tumors like medulloblastomas, embryonal tumors (primitive neuroectodermal tumors), glioblastoma have poorer prognosis than low-grade astrocytomas, gangliogliomas and meningeal tumors. One study mentioned that survival was 48.1±9.6% for patients with medulloblastoma and primitive neuroectodermal tumors; 83.3±6.2% for low-grade astrocytoma; 56.6±16.6% for ependymoma, while 0% at 72 months for high-grade glioma [19]. Literature reveals that there is very high incidence of endocrine, neurologic, and cognitive deficits in survivors [20,5]. So it is at far most important that parents should be consented and counseled regarding treatment and prognosis.

## Conclusion

Neonatal brain tumors are fortunately rare but with very high mortality and morbidity. Most of the survivors suffer from endocrinological, neurological or cognitive deficits. Parents should be informed and consented for the treatment and prognosis of the children. Cumulative approach by pediatricians, neurosurgeons should be taken in highly specialized centers with long term follow up.

## Footnotes

**Source of Support:** None

**Conflict of Interest:** None

## Figures and Tables

**Table 1:** radiological features of common neonatal brain tumors [21, 22]

Tumor type	Location	Radiological appearance
**Teratoma**	Supratentorial (common), Infratentorial (less often)	Large, solid/cystic, heterogeneous signal intensity on T1- and T2- weighted images, mixed-density lesion in CT, hypodense fatty components, coarse calcification, sometimes reflect the presence of bone and teeth
**Choroid plexus papilloma**	Supratentorial	Intraventricular mass with homogeneous contrast enhancement, Homogeneous intermediate T1 signal and high T2 signal, Hydrocephalus (common)
**Astrocytoma**	Supratentorial (optic nerve, and hypothalamic/chiasmatic region), Infratentorial (less often)	Pilocytic astrocytoma (solid component) shows hypointense on T1 and hyperintense on T2 but higher grade may show heterogenous intensity.
**Medulloblastoma (embryonal tumors)**	Infratentorial	Heterogeneous signal intensity on T1- and T2- weighted images, Curvilinear/sparse calcification
**Ganglioglioma **	Supratentorial	Microscopic calcification, Diffusion restriction not seen
**Glioblastoma**	Supratentorial	isointense to the brain or slightly hyperintense on T1-weighted images and isointense on T2-weighted images, Diffusion restriction seen
